# Effectiveness of the BBV-152 and AZD1222 vaccines among adult patients hospitalized in tertiary hospitals in Odisha with symptomatic respiratory diseases: A test-negative case–control study

**DOI:** 10.3389/fpubh.2022.1041586

**Published:** 2023-01-06

**Authors:** Debdutta Bhattacharya, Srikanta Kanungo, Subrata Kumar Palo, Jaya Singh Kshatri, Matrujyoti Pattnaik, Shishirendu Ghosal, Pranab Mohapatra, C. Mohan Rao, Avinav Sahoo, Rudra Prasanna Mishra, Sanghamitra Mishra, Amiya Ranjan Mohanta, Chinki Doley, Sanghamitra Pati

**Affiliations:** ^1^ICMR- Regional Medical Research Centre, Bhubaneswar, Odisha, India; ^2^Kalinga Institute of Medical Sciences, Bhubaneswar, Odisha, India; ^3^Hi-Tech Medical College and Hospital, Bhubaneswar, Odisha, India; ^4^Employee's State Insurance Corporation (ESIC) Hospital, Rourkela, Odisha, India; ^5^Institute of Medical Sciences & SUM Hospital, Bhubaneswar, Odisha, India

**Keywords:** BBV-152, AZD1222, COVAXIN, COVISHIELD, vaccine effectiveness

## Abstract

Two vaccines, namely BBV-152 (COVAXIN^®^) and AZD1222 (COVISHIELD™), were deployed against SARS-CoV-2 in India from January 16, 2021. Frontline health care workers were vaccinated first, followed by the adult population. However, limited data on vaccine effectiveness are available for the population of India. Therefore, we aimed to evaluate the effectiveness of two doses of each of these two common vaccines against COVID-19 infection among hospitalized patients with pulmonary conditions. We adopted a test-negative case–control design and recruited a sample of adults who were admitted to one of six tertiary care hospitals in Odisha. All participants were hospitalized patients with COVID-19-like pulmonary signs and symptoms. Participants who tested positive for SARS CoV-2 *via* RT-PCR were treated as cases, and those who tested negative were treated as controls. Logistic regression, adjusted for participants' age, sex, and number of comorbidities, was used to calculate the effectiveness of the two vaccines, using the formula: 100^*^(1 – adjusted odds ratio). Between March and July of 2021, data were collected from 1,614 eligible adults (864 cases and 750 controls). Among all participants, 9.7% had received two doses of one of the two COVID-19 vaccines. Vaccine effectiveness was 74.0% (50.5%−86.0%) for two doses of BBV-152 and 79.0% (65.4%−87.2%) for two doses of AZD1222. Thus, two doses of either BBV-152 or AZD1222 nCoV-19 vaccine were found to be substantially effective in protecting against COVID-19-related infection.

## 1. Introduction

The rapid spread of the COVID-19 pandemic elicited worldwide efforts in health care to address an urgent and essential need for effective therapeutic strategies against SARS-CoV-2. Considerable mortality and morbidity have been caused by the COVID-19 pandemic (1), which has provided a subtle reminder to the world that emerging infectious diseases can endanger lives, disrupt societies, and damage economies. Vaccines are among the most reliable and cost-effective public health interventions, reducing morbidity as well as mortality worldwide (2). Sputnik V was the first COVID-19 vaccine, developed and registered by Russia; it was followed by the Pfizer and Moderna vaccines, which were put to emergency use by the FDA of the USA (3, 4). Population studies on the effectiveness of these vaccines have been conducted in several countries (5, 6), where they have been found to be effective in providing protection against severe disease (7).

Two vaccines, AZD1222 (COVISHIELD) and BBV-152 (COVAXIN), were approved by the Indian government on 3rd January, 2021 for use in a vaccination drive. The AZD1222 vaccine was created by the University of Oxford; it employs a replication-deficient chimpanzee viral vector, based on a weakened form of an adenovirus (common cold virus) that infects chimpanzees, and carries the genetic code for the SARS-CoV-2 virus spike protein. The BBV-152 vaccine, which was developed by Bharat Biotech, is a liquid vaccine that contains whole virion inactivated SARS-CoV-2 virus. These two vaccines were initially administered to health care workers and then gradually provided to the general population, where they were observed to be effective in prevention of symptomatic COVID-19 (8). In conjunction with the continuation of the vaccination program, the effectiveness of these vaccines among the broader Indian population should be evaluated in real-world scenarios.

Vaccine effectiveness has been described as reduction in the risk of infection with or adverse effects of a disease (9). The efficacy of a vaccine under controlled conditions differs greatly from its efficacy real-world settings; hence, studies of vaccine effectiveness are essential (10) to identify the generalizability of a vaccine's effects among not only the vulnerable, but also the entire general population. Mass vaccination strategies are essential in halting the pandemic, but data on the effectiveness of vaccines are crucial in guiding future policy decisions and fostering public trust. Hence, we conducted a study based on a test-negative case–control design to evaluate separately the effectiveness of two doses of the AZD1222 or BBV-152 vaccine among hospitalized patients with COVID-19-like pulmonary diseases, with or without SARS-CoV-2 infection, which was tested by means of reverse transcription polymerase chain reaction (RT-PCR).

## 2. Methodology

### 2.1. Study design and participants

This study was based on a test-negative case–control design, and the participants were patients who were admitted to tertiary care hospitals in four cities in Odisha, namely Bhubaneswar, Puri, Rourkela, and Bolangir. Studies using the test-negative case–control design have been found to be sufficiently powerful to estimate the effectiveness of vaccines against various respiratory diseases; such studies have also been found to exhibit a high level of agreement with the findings of randomized controlled studies (11–13). As per the “Evaluation of COVID-19 vaccine effectiveness” guidelines, a set of recommendations for vaccine efficacy studies published by the World Health Organization (14), the participants recruited for this study were hospitalized patients with signs and symptoms of pulmonary diseases similar to those of SARS-CoV-2, such as sore throat, cough, bronchitis, breathlessness, pneumonia accompanied by fever, headache, or body aches.

### 2.2. Case definition

Patients having symptomatic pulmonary disease and positive confirmation of SARS-CoV-2 infection *via* RT-PCR were regarded as “cases” (test-positive). In contrast, “controls” (test-negative) were those patients who had signs and symptoms of pulmonary disease but tested negative *via* RT-PCR for SARS-CoV-2 infection.

### 2.3. Inclusion and exclusion criteria

Individuals were included in the study if they were eligible to receive any of the vaccines (aged ≥18 years) during the study period, were able to provide informed consent, and were admitted to the health care facility with symptomatic respiratory illness. Between March 2021 and July 2021, 16,827 patients were admitted across the six tertiary hospitals in Odisha. Twelve thousand and sixty-five patients were excluded due to having no signs and symptoms of respiratory disease or clinical diagnosis report. Of the remaining 4,762 patients, 1,954 individuals were excluded due to being under 18 years of age. Any participants exhibiting clinical symptoms of the disease of interest (here COVID-19) within 2 weeks before vaccination cannot participate in a vaccine efficacy study (14); for this reason and on the basis of other exclusion criteria, such as readmission or not meeting the case criteria, another 1,194 individuals were excluded from the final sample. Following these exclusions, 1,614 individuals formed the final sample for the study, with 864 being cases and 750 being controls.

### 2.4. Sample size

The formula used for sample size calculation was:


$$\begin{array}{l} \text{N}1\;=\;{\left(\text{z}/\text{d}\right)}^{2}\;\left[1/\text{A }\left(1-\text{A}\right)\;+\;1/{\text{C}}_{2}\;\left(1-{\text{P}}_{2}\right)\right] \end{array}$$

Based on a minimum of one control per case, a specified precision of ± 10%, and a type I error rate of 0.05, and assuming vaccine effectiveness of 50% and coverage of 50%, the required sample size was calculated to be:

Cases: 828 & Controls: 828

Although we identified sufficient cases, as per the case definition, the hospital setting did not quite provide sufficient eligible controls to achieve the specified sample size.

### 2.5. Data collection

Data were collected in the same way for both cases and controls. Clinical data for each individual were obtained from the patient records (both electronic and print) of the relevant hospital. A range of data were collected from the records, including name, age, gender, ethnicity, phone number, place, occupation, presence of comorbidities (asthma, hypertension, diabetes, chronic renal diseases, chronic obstructive pulmonary diseases, cardiovascular diseases, sickle cell anemia, rheumatoid diseases, etc.), COVID-19 testing data, symptoms, oxygen requirements during admission, and outcome. Vaccination data for each patient in the study were obtained by checking their vaccination certificate and hospital records. In cases of unavailability of this data, the patient or their caregiver was contacted for this information. To avoid observer bias, a deidentified dataset was constructed for data analysis and provided to researchers other than those who were involved in data collection.

### 2.6. Statistical analysis

Descriptive statistics on vaccination status are presented in the form of frequencies (*n*, %); continuous variables (age, years of education) are summarized in terms of mean (±standard deviation) and/or median with interquartile range (IQR). For the purpose of COVID-19 vaccine effectiveness studies, an individual is considered to be potentially protected as a result of their vaccination only once 14 days have elapsed since their first dose and 7–14 days have elapsed since their second dose, if applicable (14). In accordance with these guidelines, participants were categorized into three groups based on the number of days that had passed since their second vaccine dose (“ <14 days,” “14–28 days,” or “more than 28 days”) as a measure of their vaccine coverage status (see Table 1). Vaccine effectiveness was calculated in terms of odds ratio (OR) using the formula: 100^*^(1-OR), and is reported along with a 95% confidence interval.

**Table 1:** Vaccination status among cases and controls.

**Vaccine type**	**Number of doses**	**Days from vaccination to enrollment in study**	**Cases**	**Controls**	**Total**

			***n*** **(%)**	***n*** **(%)**	***n*** **(%)**
COVAXIN (BBV-152)	Double dose	<14 days	4 (26.7)	22 (75.9)	26 (59.1)
		14–28 days	3 (20.0)	2 (6.9)	5 (11.3)
		>28 days	8 (53.3)	5 (17.2)	13 (29.6)
COVISHIELD (AZD1222)	Double dose	<14 days	12 (50.0)	31 (52.5)	43 (51.8)
		14–28 days	7 (29.2)	4 (6.8)	11 (13.3)
		>28 days	5 (20.8)	24 (40.7)	29 (34.9)

Logistic regression, using both adjusted and unadjusted methods, was used to compare the proportion of individuals testing positive among those who had received two doses of either vaccine to the proportion among those who had not been vaccinated. Data from the same sample of unvaccinated participants were entered into the comparison for each vaccine. Observations were entered into the regression model only for participants for whom the number of doses received, vaccine received (BBV-152 or AZD1222), and RT-PCR test result had all been recorded. The effectiveness of each vaccine was analyzed separately. Potential confounders and biases, such as age, gender, and number of comorbidities that might influence vaccine effectiveness, were adjusted for in the analysis.

The statistical software package STATA (v. 16.0; StataCorp LLC, Texas, USA) was used for data cleaning and statistical analyses. A *p*-value of < 0.05 was taken as the threshold for significance.

## 3. Results

The median age of participants with confirmed SARS-CoV-2 infection was found to be 50 years, with an IQR of 40–60 years. Among these, 72.8% were men and more than one-third of them fell into the 45–60 age bracket (Table 2). The median age among the controls was 45 years (IQR: 30–59 years), of whom 68.1% were men and nearly one-fourth of them fell into the same age bracket as the cases (45–60 years). The majority of both cases (62.6%) and controls (56.8%) were residents of rural Odisha. Among participants who were diagnosed with COVID-19 infection, 5.9% were health care workers. The prevalence of comorbidities was lower among the control group (23.0%) than among those who were diagnosed with COVID-19 (63.0%).

**Table 2:** Baseline characteristics across cases (*n* = 864) and controls (*n* = 750).

**Characteristics**	**Cases *n* (%)**	**Controls *n* (%)**	**Total**
Age (years): median (IQR)	50 (40–60)	45 (30–59)	47.5 (35–60)
Age (years): mean ± SD	50.0 ± 14.1	45.2 ± 17.0	47.8 ± 15.7
**Age group (in years) (*****n*** = **1,614)**			
18–29	52 (6.0)	174 (23.2)	226 (14.0)
30–44	254 (29.4)	198 (26.4)	452 (28.0)
45–59	331 (38.3)	192 (25.6)	523 (32.4)
60 and above	227 (26.3)	186 (24.8)	413 (25.6)
**Gender (*****n*** = **1,614)**			
Male	629 (72.8)	511 (68.1)	1,140 (70.6)
Female	235 (27.2)	239 (31.9)	474 (29.4)
**Area of residence (*****n*** = **1,614)**			
Rural	541 (62.6)	426 (56.8)	967 (59.9)
Urban	323 (37.4)	324 (43.2)	647 (40.1)
**Caste (*****n*** = **1,614)**			
General	471 (54.5)	409 (54.5)	880 (54.5)
OBC	252 (29.2)	260 (34.7)	512 (31.5)
SC	74 (8.6)	22 (3.0)	96 (6.2)
ST	67 (7.7)	59 (7.8)	126 (7.8)
**Education (*****n*** = **1,614)**			
Years of schooling	11.4 ± 3.7	11.7 ± 3.7	11.5 ± 3.7
**Occupation (*****n*** = **1,614)**			
Health care workers	23 (2.6)	26 (3.5)	49 (3.1)
Front line workers	28 (3.3)	31 (4.1)	59 (3.6)
Others	813 (94.1)	693 (92.4)	1,506 (93.3)
**Number of comorbidities (*****n*** = **1,614)**			
0	320 (37.0)	580 (77.3)	900 (55.7)
1	379 (43.9)	103 (13.7)	482 (29.9)
2	132 (15.3)	49 (6.6)	181 (11.2)
3 or more	33 (3.8)	18 (2.4)	51 (3.2)

At the time at which this study was conducted, 9.7% of the study population (157 out of 1,614) had been vaccinated with two doses of either of the vaccines. Among these, 59 had received two doses of BBV-152 and the other 98 patients had received two doses of AZD1222. However, data on both date of vaccination and details of hospitalization were available for only 44 (74.6%) and 83 (84.7%) of the participants in the BBV-152 and AZD1222 groups, respectively.

Among the 44 participants who had received two doses of BBV-151, 26 (59.1%) were admitted to the hospital within 2 weeks of receiving their second dose; another 5 (11.3%) were admitted within 2–4 weeks, and 13 (29.6%) >4 weeks later. Of patients who had received both doses of AZD1222, 28 (28.6%) were considered to be cases and 70 (71.4%) controls, as per their RT-PCR results. Among the 24 cases for whom the dates of second vaccine dose and hospitalization were both available, 12 (50.0%) were hospitalized with respiratory distress within 2 weeks of their second dose, while a smaller number were admitted beyond this period [7 (29.2%) were hospitalized within 14–28 days, and 5 (20.8%) were admitted at least 4 weeks after receiving their second dose].

The effectiveness of each of the two vaccines, AZD1222 and BBV-152, is presented in Table 3. Unadjusted vaccine effectiveness for a double dose of AZD1222 was calculated to be 70.2% (53.1%−81.0%), and for BBV-152, unadjusted effectiveness was 65.0% (38.9%−80.0%). Adjusted vaccine effectiveness was calculated to be 79.0% (65.4%−87.2%) and 74.0% (50.5%−86.0%) for a double dose of AZD1222 and BBV-152, respectively.

**Table 3:** Vaccine effectiveness (VE) for double doses of the BBV-152 and AZD1222 vaccines.

**Vaccination status**	**COVISHIELD (AZD1222)**				**COVAXIN (BBV-152)**			
	**Cases**	**Controls**	**VE (95% CI)**	**Adj. VE** ^*^ **(95% CI)**	**Cases**	**Controls**	**VE (95% CI)**	**Adj. VE** ^*^ **(95% CI)**
Unvaccinated	713 (57.3)	532 (42.7)	Ref.	Ref.	713 (57.3)	532 (42.7)	Ref.	Ref.
Double dose	28 (28.6)	70 (71.4)	70.2% (53.1%−81.0%)	79.0% (65.4%−87.2%)	19 (32.2)	40 (67.8)	65.0% (38.9%−80.0%)	74.0% (50.5%−86.0%)

* Adjusted for age, sex, and number of comorbidities.

## 4. Discussion

The vaccine effectiveness of two doses of AZD1222 or BBV-152 was tested among adults (>18 years) with a range of comorbid conditions. Two doses of AZD1222 were found to have 79% effectiveness, while the effectiveness of the BBV-152 vaccine was 74.0%. These findings indicate that the effectiveness of the AZD1222 and BBV-152 vaccines in averting COVID-19 infection among hospitalized adults in Odisha with respiratory symptoms is generally high.

The adjusted vaccine effectiveness of two doses of BBV-152 observed in our study was 74.0% (95% CI: 50.5%−86.0%), which is close to the efficacy of the vaccine against symptomatic COVID-19 disease observed during its phase 3 clinical trials, namely 77.8% (95% CI: 65.2%−86.4%) (15). Furthermore, the vaccine effectiveness of two doses of AZD1222 against symptomatic COVID-19 was found to be 79.0% (95% CI: 65.4%−87.2%), which is higher than the efficacy of the same vaccine (70.4%) as evaluated based on a pooled analysis of four randomized, double-blind controlled trials (16). Thus, the results for both the vaccines were similar to those attained in their phase 3 clinical trials.

The effectiveness of the AZD1222 and BBV-152 vaccines has been calculated in many studies. A study among HCWs in the armed forces of India vaccinated with the AZD1222 vaccine reported a 91%−94% reduction in risk of breakthrough cases of COVID-19 (17). Hospitalization was found to be reduced by about 88% due to vaccination in a cohort study conducted in Scotland (16, 18). Various other studies have shown a 60%−70% reduction in breakthrough infections of vaccinated individuals (7, 16, 19), which supports the findings of our study.

This study has several strengths of its own. First, a suitable level of power was achieved through recruitment of a sample of scientifically calculated size that included all adult age groups in the community, recruited since the beginning of the vaccination program among the general population. The evidence generated on vaccine effectiveness is highly generalizable due to the variability of the sample characteristics (i.e., data were collected from various cities and hospitals without restricting the sample to specific populations or communities). Adjusting for various covariates in the regression model somewhat reduced the risk of bias attributable to vaccination status or COVID-19 infection rates.

The limitations of the study are attributable to its observational nature, meaning that its results must be interpreted with caution. Erroneous handling of RT-PCR samples tested in various labs might have resulted in false reports, which may have produced some degree of error in the vaccine effectiveness observed in the study in an unknown direction. Additionally, this was a hospital-based study, and the majority of mild cases occurring during the second wave of the pandemic did not result in hospital admissions, which might have caused discrepancy in the observed effectiveness of both the vaccines. Furthermore, non-uniformity of hospital admission policies with respect to patients' clinical condition might have exerted an effect on the vaccine effectiveness observed. Finally, differences in time course between vaccinated and unvaccinated individuals, occurring due to various factors, may have created biases; these potential confounders can better be assessed and interpreted with the help of a large cohort study.

## 5. Conclusion

In conclusion, the study findings will aid formulation of a strategy to develop maximum utilization of the AZD1222 and BBV-152 vaccines in public health practice. The findings provide assurance on the advantages of deploying these vaccines, and on the necessity of administering two doses, particularly among populations where the incidence of SARS-CoV-2 infection remains high.

## Data availability statement

The raw data supporting the conclusions of this article will be made available by the authors, without undue reservation.

## Ethics statement

The studies involving human participants were reviewed and approved by Institutional Ethical Committee of ICMR—Regional Medical Research Centre, Bhubaneswar. The patients/participants provided their written informed consent to participate in this study.

## Author contributions

SP designed the study and critically reviewed the manuscript. DB, SK, PM, CR, AS, RM, SM, AM, and CD performed coordination, data collection, and management. JSK, SKP, SK, SG, and MP carried out the data analysis. MP wrote the manuscript. All authors read and approved the final manuscript.
